# Explaining decisions of a light-weight deep neural network for real-time coronary artery disease classification in magnetic resonance imaging

**DOI:** 10.1007/s11554-023-01411-7

**Published:** 2024-02-10

**Authors:** Talha Iqbal, Aaleen Khalid, Ihsan Ullah

**Affiliations:** 1 Insight SFI Research Centre for Data Analytics, University of Galway, Galway, H91 TK33 Ireland; 2 School of Computer Science, University of Galway, Galway, H91 TK33 Ireland

**Keywords:** Healthcare models, Time complexity, Hyper-parameter tuning, Explainable AI, Classification

## Abstract

In certain healthcare settings, such as emergency or critical care units, where quick and accurate real-time analysis and decision-making are required, the healthcare system can leverage the power of artificial intelligence (AI) models to support decision-making and prevent complications. This paper investigates the optimization of healthcare AI models based on time complexity, hyper-parameter tuning, and XAI for a classification task. The paper highlights the significance of a lightweight convolutional neural network (CNN) for analysing and classifying Magnetic Resonance Imaging (MRI) in real-time and is compared with CNN-RandomForest (CNN-RF). The role of hyper-parameter is also examined in finding optimal configurations that enhance the model’s performance while efficiently utilizing the limited computational resources. Finally, the benefits of incorporating the XAI technique (e.g. GradCAM and Layer-wise Relevance Propagation) in providing transparency and interpretable explanations of AI model predictions, fostering trust, and error/bias detection are explored. Our inference time on a MacBook laptop for 323 test images of size 100x100 is only 2.6 sec, which is merely 8 milliseconds per image while providing comparable classification accuracy with the ensemble model of CNN-RF classifiers. Using the proposed model, clinicians/cardiologists can achieve accurate and reliable results while ensuring patients’ safety and answering questions imposed by the General Data Protection Regulation (GDPR). The proposed investigative study will advance the understanding and acceptance of AI systems in connected healthcare settings.

## Introduction

According to the World Health Organization^1^, in 2019, an estimated 17.9 million people died from cardiovascular diseases, representing 32% of all global deaths. Statistics published by the American Heart Association in 2023 state that from 2017-2020, an estimated 20.5 million Americans had coronary heart disease (CHD) [1]. Specifically, Coronary artery disease (CAD) accounts for approximately 610,000 deaths annually in the United States and is the third leading cause of death worldwide, with 17.8 million deaths annually [2].

The patient’s symptoms of CAD are neither sensitive nor specific, thus making it difficult for clinicians or cardiologists to rely only on them. The reference standard for CAD detection is coronary angiography, which is an invasive diagnostic imaging procedure performed using cardiac catheterization [3]. This method is expensive and carries potential risks. Other methods include cardiac imaging techniques, which are safe, non-invasive, cheaper and can help doctors in early detection and providing timely interventions to treat CAD patients. These techniques include X-rays, Computer Tomography (CT), Echo-cardiogram and Magnetic Resonance Imaging (MRI) or Cardiac Magnetic Resonance (CMR) Imaging [4].

X-rays and CT imaging techniques use ionizing radiations, which are considered harmful if a patient is over-exposed to them [5]. Echocardiograms are limited by cost, time, and acoustic window access [6]. MRI or CMR imaging uses magnetic waves and is considered a viable alternative for non-invasive assessment of CAD [7]. MRI/CMR images provide precise measurements of heart structure and functions, as well as myocardial perfusion and parametric quantification. MRI/CMR could be 2D or 3D, but 3D imaging has excessive artifacts and has thus not been clinically used for the diagnosis of CAD [8]. Manual interpretation of 2D scans is also time-consuming and requires experience. Thus, artificial intelligence methods are exploited to automate the CAD diagnosis to reduce the analysis time with potentially improved accuracy. This plays a critical role in connected healthcare settings (transitioning healthcare services remotely, from hospitals to patient side or home-based care).

However, there are several challenges in implementing such AI models on computational tools such as Field Programmable Gate Arrays (FPGAs), Raspberry Pi and central processing unit (CPU)/graphics processing unit (GPU) based systems. These challenges arise due to the limited processing power, memory, and energy efficiency of these devices. It is essential to engage in a multidisciplinary approach that involves collaboration between domain experts, data scientists and hardware engineers to overcome these challenges.

Convolutional Neural Network (CNN) models have yielded unprecedented achievements in addressing computer vision challenges, including but not limited to image classification, object detection, and tracking. Nonetheless, their integration into embedded applications has been impeded by the substantial computational and memory requisites, thereby giving rise to a novel research domain known as model compression including bit reduction, knowledge distillation, tensor decomposition, network pruning, and microarchitecture [9]. Interested readers are referred to [10] for detailed insights, advantages and limitations of each mentioned method. While these strategies have demonstrated notable achievements, they are not without their inherent constraints.

This paper introduces a lightweight Convolutional Neural Network (CNN) model designed specifically for real-time implementation as a classifier. In connected healthcare settings, where low latency and efficient processing are crucial, this lightweight CNN offers a promising solution. By optimizing the model’s architecture and parameters, we aim to strike a balance between computational efficiency and classification accuracy, enabling real-time CAD detection. This approach has great potential to improve the deployment of AI systems in resource constrained environments, ultimately benefiting the overall healthcare systems.

The remaining paper is organised as; Sect. 2 summaries the available literature on real-time CAD classification networks, Sect. 3 highlights the proposed work and dataset description, Sect. 4 provides calculations and the experimental results and the conclusion and future work are presented in Sect. 6.

## Background

Coronary artery disease (CAD) primarily originates from the accumulation of atherosclerotic plaque within the epicardial arteries, leading to an imbalance in the supply and demand of oxygen to the myocardium, often resulting in ischemia [11]. Chest pain is the predominant symptom, typically occurring during physical or emotional stress. Lifestyle modifications, pharmacological therapies, and invasive interventions are available strategies to modify this disease process, with the goal of stabilizing or regression of the disease [12]. Despite the development of innovative imaging methods, such as MRI and/or coronary CT angiography, invasive coronary angiography remains the preferred diagnostic tool for assessing the severity of complex CAD, as endorsed by the 2019 guidelines of the European Society of Cardiology [13]. The process of interpreting complex coronary vascular structures is a time-intensive task and presents challenges to the clinician [14]. The implementation of real-time automatic CAD detection and labelling offers promise in overcoming these challenges by providing valuable support in the decision-making process.

Numerous methodologies for the automatic or semi-automatic assessment of coronary artery diseases have been proposed by various research groups [15]. These methodologies adhere to a common framework comprising three fundamental steps: (1) extraction of the coronary artery tree, (2) computation of geometric parameters, and (3) analysis of stenotic segments. The pivotal phase significantly influencing the efficiency and precision of these algorithms is dependent on the extraction of the coronary artery tree. This task is accomplished through diverse techniques, including centerline extraction [16], graph-based methods [17], superpixel mapping [18], and machine/deep learning [19]. Among these, machine and deep learning methods have exhibited substantial potential in CAD detection based on their commendable performance, adaptability to tuning, and optimization capabilities [20]. The overarching objective pursued by developers and users of CNNs is to strike an optimal equilibrium between accuracy and speed, a concept often referred to as the “speed/accuracy trade-off” [21]. This trade-off incorporates the endeavour to achieve high levels of CAD detection accuracy while simultaneously ensuring swift processing and analysis, a critical consideration in clinical practice.

Although several CNN-based approaches have reportedly achieved optimal accuracy in CAD detection, with Dice Similarity Coefficients surpassing 0.75 [22] and Sensitivity metrics exceeding 0.70 [23], there is notable neglect of their processing speed. The time required for image processing represents a critical performance indicator for the practical application of these methods. In literature, studies have reported processing times ranging from 1.1 to 11.87 seconds [17, 22], 20 seconds [18] and, in some instances, even exceeding 60 seconds per frame [16]. However, such durations are considered unacceptable for real-time CAD detection as the required processing time is 0.13 to 0.07 seconds per frame [24]. Thus, our study presents a detailed analysis of light-weighted neural network architectures along with their potential in terms of accuracy and performance to classify healthy and CAD images.

## Proposed work

**Fig. 1:**
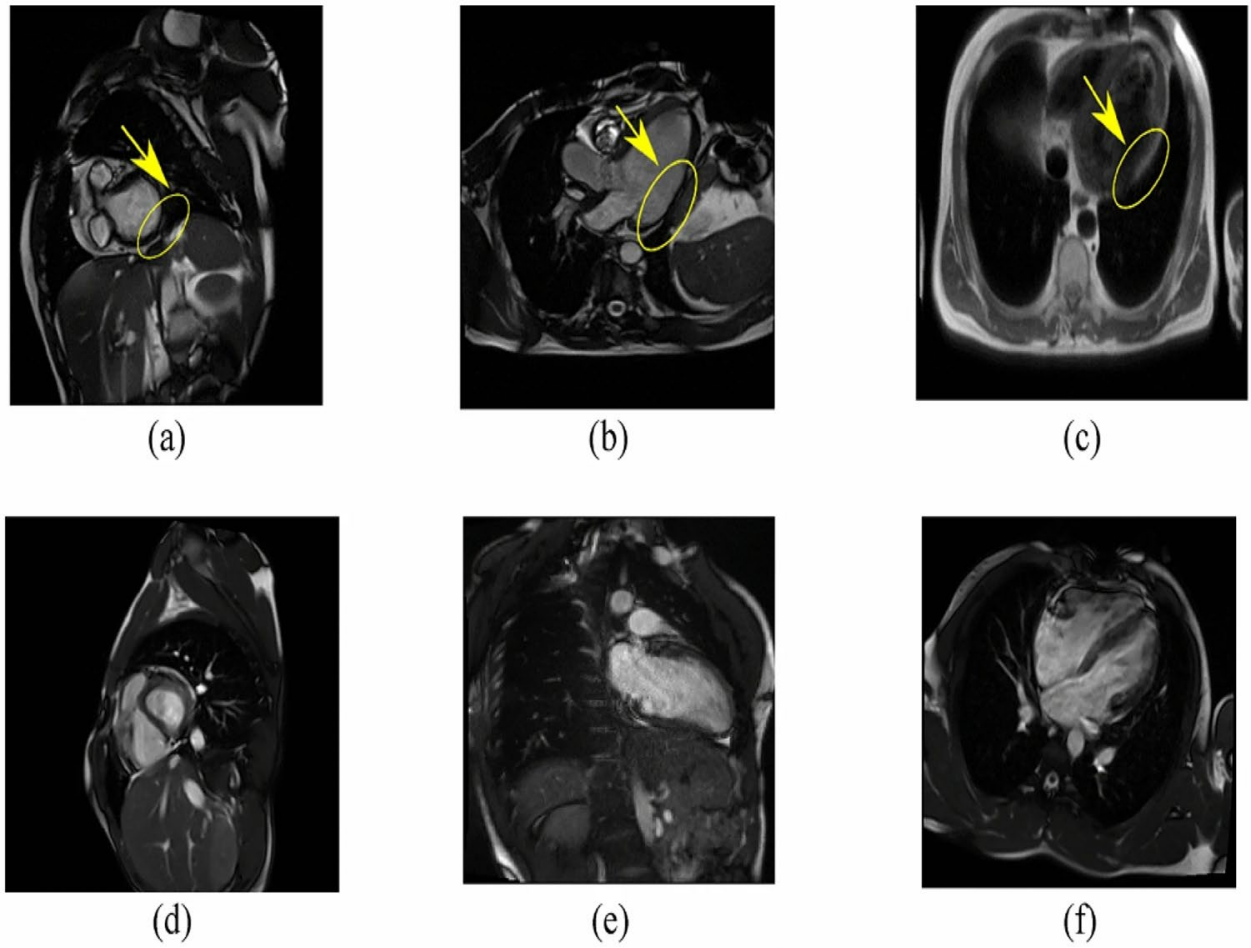
Example of 2D MRI/CMR images from CAD patients (**a**–**c**) and healthy subjects (**d**–**f**). The yellow circle highlights the region indicative of CAD in sub-images (**a**–**c**). Figure reproduced with permission from [26]

In the proposed work, we implemented a light-weight neural network, that is, adapted version of LeNET-5 model [25] on the CAD Cardiac Magnetic Resonance Imaging dataset^2^ (proposed by Khozeimeh *et al.* [26]) for a comprehensive comparison. The results of the CNN-RF model [26] were considered as ground truth/reference. The input to the model is 2D CMR images. Figure 1 depicts examples of both categories’ images. Pre-processing steps included resizing the images to 100x100 pixels and normalization between 0 and 1. The main contribution in the proposed work is 3-fold and is as follows:

1. Time Complexity: We propose a lightweight deep network model for CAD classification in MRI by carefully selecting network architecture and optimizing model parameters to reduce inference time while maintaining accuracy and enabling real-time or near-real-time CAD diagnosis.
2. Hyper-parameter Tuning: We optimized the deep model by exploring different hyper-parameter settings as well as used various activation functions, optimizers, and architectural changes, to identify configurations that maximize the model’s performance.
3. eXplainable Artificial Intelligence (XAI): We integrated GradCam [27] and Layer-Wise Relevance Propagation (LRP) [28], XAI techniques to provide interpretable insights into the model’s decision-making process, generating heatmaps that highlight the regions of MRI/CMR images that govern CAD classification.

### Dataset description

The dataset consists of 63,151 multiparametric CMR Images including 37,290 healthy and 25,861 CAD patients images. CAD diagnosis was confirmed by invasive coronary angiography. Four MRI / CMR sequences (that is, Late Gadolinium Enhancement (LGE), Perfusion, T2 weighted, and Steady-State Free Precession (SSFP)) were used, capturing short and long axes plains of the heart. A total of 13 slices per patient were collected in four types of sequences.

During the pre-processing stage, a manual inspection was conducted on images from both subsets, and any images with poor MRI/CMR quality were excluded from further analysis. Following the pre-processing stage, the dataset consisted of 34,216 images from healthy patients and 17,438 images from patients with CAD.

### Performance assessment matrices

The performance of the classifier is assessed using Positive Predictive Value (PPV), Recall (Sensitivity or True Positive Rate), Specificity (True Negative Rate), F1-Score, Area Under the Curve (AUC), Accuracy and Balanced Accuracy. Mathematically, each matrix is presented as:

1 $$\begin{aligned}{} & {} \text {PPV} = \frac{\text {TP}}{\text {TP} + \text {FP}} \end{aligned}$$

2 $$\begin{aligned}{} & {} \text {Recall} = \frac{\text {TP}}{\text {TP} + \text {FN}} \end{aligned}$$

3 $$\begin{aligned}{} & {} \text {Specificity} = \frac{\text {TN}}{\text {TN} + \text {FP}} \end{aligned}$$

4 $$\begin{aligned}{} & {} \text {F1-score} = 2 \cdot \frac{\text {Precision} \cdot \text {Recall}}{\text {Precision} + \text {Recall}} \end{aligned}$$

5 $$\begin{aligned}{} & {} \text {AUC} = \int _{0}^{1} \text {ROC-curve} \, d\text {FPR} \end{aligned}$$

6 $$\begin{aligned}{} & {} \text {Accuracy} = \frac{\text {TP} + \text {TN}}{\text {TP} + \text {TN} + \text {FP} + \text {FN}} \end{aligned}$$

7 $$\begin{aligned}{} & {} \text {Balanced Accuracy} = \frac{\text {Sensitivity} + \text {Specificity}}{2} \end{aligned}$$

Where *TP:* True Positives, *TN:* True Negatives, *FP:* False Positives,* FN:* False Negatives, *ROC:* Receiver Operating Characteristic and *FPR:* False Positive Rate.

## Results

**Fig. 2:**
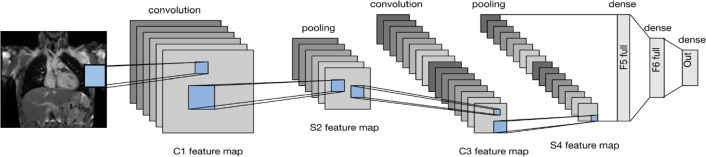
Implemented model architecture

Figure 2 illustrates the implemented model architecture^3^. All experiments were implemented in Python using the Karas library. The models were trained on Apple M2 Pro with 16 GB RAM. The following subsections discuss the time complexity calculations, the effect of hyper-parameter tuning, and feature explanations using XAI results.

### Time complexity calculation and comparison

The time complexity of a model is determined by the number of layers and the operations performed in each layer. The proposed model architecture comprises seven layers, excluding the input layer, as shown in Fig. 2. These layers consist of C1 (convolutional), S2 (subsampling), C3 (convolutional), S4 (subsampling), FC5 (fully connected), FC6 (fully connected) and the output layer. The time complexity of each layer is as follows:

1. C1 (convolutional layer): The time complexity of this layer depends on the filter size, the shape of the input image, and the number of filters applied. Assuming the input shape is (H, W, C), where H is the height, W is the width, and C is the number of channels, and the filter size is (FH, FW), the time complexity is approximately O(H * W * C * FH * FW * F *$S^{2}$), where F is the number of filters and S is stride value.
2. S2 (subsampling layer/ average pooling layer): The time complexity of this layer depends on the pool size and strides. Assuming that the pool size is (PH, PW) and the strides are (SH, SW), the time complexity is approximately O((H/PH) * (W/PW) * C).
3. C3 (convolutional layer): Similar to C1, the time complexity of this layer is approximately O(H * W * C * FH * FW * F * $S^{2}$).
4. S4 (subsampling layer/ average pooling layer): Similar to S2, the time complexity of this layer is approximately O((H/PH) * (W/PW) * C).
5. FC5 (fully connected layer): The time complexity of a fully connected layer with units U is O(U * F), where F is the size of the input features.
6. FC6 (fully connected layer): The time complexity of this layer is similar to the previous one, that is, O(U * F).
7. Output layer: The time complexity of the output layer is O(U), where U is the number of output units.

Adding all the time complexities of each layer, the overall time complexity of the proposed model could be approximated to be: $O\Big (2*(H * W * C * FH * FW * F * S^{2}) + \frac{H}{PH} * \frac{W}{PW} * C) + (ep * ts * tf * C * fs * fs) + 2*( U * F) + U\Big ).$

In our case, the input image shape is (100,100,1), filter size is varied between (C1 = 6, C3 = 6) and (C1 = 12, C3 = 6), kernel size = (5,5), pooling size = (2,2), strides = (2,2), units in fully connected layer 1 and layer 2 = 128, 84 respectively, while the output layer had only 1 unit, as the model is performing binary classification.

As the results are to be compared with CNN-RF models proposed by [26], the time complexity of their model is calculated to be: $O\big (n_{\text {e}} \log (n_{\text {e}}) n_{\text {f}} n_{\text {s}} \log (n_{\text {s}}) + 2 \big (O(n_{\text {s}}) + O(n_{\text {cnn}} n_{\text {ep}} n_{\text {ts}} n_{\text {tf}} n_{\text {tc}} \text {fs} \text {fs}) + O(n_{\text {ts}} n_{\text {cnn}}) + O(n_{\text {ts}} n_{\text {cnn}} \log (n_{\text {cnn}})) + O(n_{\text {vs}} n_{\text {cnn}})\big )$.

In both the time complexity equations, e is estimators, f is features, s is samples, ep is epochs, ts is train samples, tf is train features, tc is train channels, fs is filter size, and vs is validation samples.

A comparison of the time complexities between the proposed model and the CNN-RF model reveals that our model entails significantly lower computational overhead in comparison to the CNN-RF model. Our inference time on a MacBook laptop for 323 test images of size 100x100 is only 2.6 sec, which is only 8 milliseconds per image. Additionally, it provides better or equal classification accuracy. Our model’s lower computational complexity enables faster image analysis and diagnosis, improving efficiency, and facilitating deployment on resource-constrained systems such as Raspberry Pi, FPGA or any other edge device for real-time classification and diagnosis in connected healthcare settings.

### Hyper-parameter tuning and classification results

Various hyperparameter configurations were utilized to attain optimal model performance for CAD image classification. Table 1, 2, 3 and 4 present the diverse performance of the models obtained with different settings.

**Table 1:** Model parameters settings: model with filter size $C_{1}$ and $C_{3}$ = 6,6; Batch Size = 32; epochs = 20; loss function = binary cross-entropy; final layer activation function = sigmoid; dropout = 0.5

Activation	Optimizer	PPV	Recall	Specificity	F1-Score	AUC	Accuracy	Balanced Acc
**PReLU**	Adam	99.33	97.83	99.66	98.57	99.90	99.04	98.75
	**RMSprop**	**98.60**	**98.77**	**99.28**	**98.67**	**99.76**	**99.11**	**99.03**
ReLU	Adam	99.21	96.85	99.61	98.02	99.84	98.67	98.23
	RMSprop	98.12	97.31	99.05	97.72	99.63	98.46	98.18
LeakyReLU	Adam	97.99	97.40	99.00	97.69	99.83	98.44	98.20
	RMSprop	98.48	98.05	99.22	98.27	99.75	98.83	98.64

**Table 2:** Model parameters settings: model with filter size $C_{1}$ and $C_{3}$ = 12,6; Batch Size = 32; epochs = 20; loss function = binary cross-entropy; final layer activation function = sigmoid; dropout = 0.5

Activation	Optimizer	PPV	Recall	Specificity	F1-Score	AUC	Accuracy	Balanced Acc
PReLU	Adam	99.65	97.45	99.82	98.54	99.86	99.02	98.64
	RMSprop	99.47	97.34	99.74	98.39	99.82	98.93	98.54
**ReLU**	**Adam**	**99.10**	**98.17**	**99.55**	**98.63**	**99.87**	**99.08**	**98.86**
	RMSprop	98.87	97.48	99.43	98.17	99.57	98.77	98.46
LeakyReLU	Adam	98.12	98.40	99.03	98.26	99.87	98.82	98.72
	RMSprop	98.61	97.14	99.30	97.87	99.74	98.57	98.22

**Table 3:** Model parameters settings: model with filter size $C_{1}$ and $C_{3}$ = 6,6; Batch Size = 32; epochs = 20; loss function = binary cross-entropy; final layer activation function = sigmoid; No dropout

Activation	Optimizer	PPV	Recall	Specificity	F1-Score	AUC	Accuracy	Balanced Acc
**PReLU**	Adam	99.19	98.03	99.59	98.60	99.88	99.06	98.81
	**RMSprop**	**99.62**	**98.45**	**99.81**	**99.04**	**99.92**	**99.35**	**99.13**
ReLU	Adam	99.33	97.28	99.66	98.29	99.90	98.86	98.47
	RMSprop	96.18	98.94	98.00	97.55	99.65	98.32	98.47
LeakyReLU	Adam	99.01	97.14	99.50	98.06	99.85	98.70	98.32
	RMSprop	98.58	97.57	99.28	98.07	99.79	98.70	98.43

**Table 4:** Model parameters settings: model with filter size $C_{1}$ and $C_{3}$ = 12,6; batch size = 32; epochs = 20; loss function = binary cross-entropy; final layer activation function = sigmoid; No dropout

Activation	Optimizer	PPV	Recall	Specificity	F1-Score	AUC	Accuracy	Balanced Acc
PReLU	Adam	99.08	98.08	99.53	98.58	99.88	99.04	98.81
	RMSprop	99.10	98.03	99.55	98.56	99.80	99.03	98.79
**ReLU**	**Adam**	**99.31**	**98.17**	**99.65**	**98.73**	**99.92**	**99.15**	**98.91**
	RMSprop	97.91	99.03	98.92	98.46	99.76	98.95	98.97
LeakyReLU	Adam	98.64	97.65	99.31	98.15	99.79	98.75	98.48
	RMSprop	98.98	97.25	99.49	98.11	99.81	98.73	98.37

The Parametric Rectified Linear Unit (PReLU) activation function combined with the Root Mean Squared propagation (RMSprop) optimizer resulted in the highest classification accuracy, achieving a general accuracy of 99.35% and a balanced precision of 99.13%. This surpasses the previously achieved highest accuracy of 99.18% obtained by the reference CNN-RF model. To test the generalizability of our model, a stratified cross-validation (CV) analysis was performed using 10-folds. The model showed similar performance as without CV, achieving classification accuracy of 99.22% (while the balance accuracy of 99.10%), as depicted in Table 5.

**Table 5:** Model’s best performances achieved with different settings: comparison

Model	Act function	Optimizer	PPV	Recall	Specificity	F1-Score	AUC	Accuracy
Our Model	PReLU	Adam	99.19	98.03	99.59	98.60	99.88	99.06
**Our Model**	**PReLU**	**RMSprop**	**99.62**	**98.45**	**99.81**	**99.04**	**99.92**	**99.35**
$Our Model^{*}$	PReLU	RMSprop	99.11	98.59	99.55	98.85	99.86	99.23
Our Model	ReLU	Adam	99.31	98.17	99.65	98.73	99.92	99.15
CNN-RF	ReLU	Adam	100	98.88	99.66	99.70	99.00	99.18

Note: ^∗^are model’s results with 10-fold Stratified Cross-Validation

**Fig. 3:**
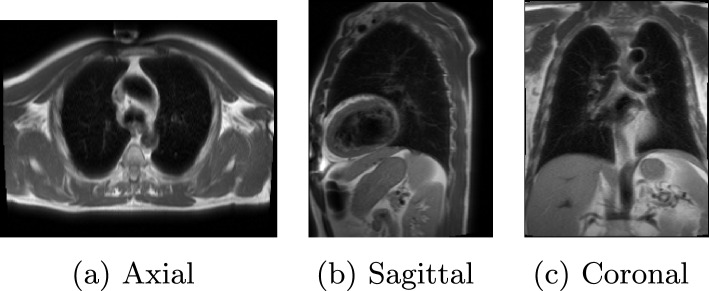
Original images from the sick dataset. **a** is Axial-view **b** is Sagittal-view while **c** shows a Coronal view of a chest MRI scan (one frame)

The sub-optimal performance of the proposed classifiers can be attributed to their reliance on the frame-based analysis. MRI sequences often produce a multitude of frames, some of which lack noticeable regions of interest (ROIs), as depicted in Figure 3 (all three view angles of MRI scan). The figure illustrates the frames with no ROIs (no visible coronary artery in the frame). The proposed model considers all the frames uniformly, irrespective of their diagnostic value. Thus, frames without ROIs introduce noise into the analysis, impairing the classifier ability to differentiate between images of patients with CAD (illness) and those of healthy individuals. This limitation underscores the need for more sophisticated methodologies that account for the inherent variability in MRI frames, enabling classifiers to consider frames based on the presence or absence of ROIs.

## eXplainable AI

Explainable Artificial Intelligence (XAI) is a field in machine learning and artificial intelligence that focuses on developing models that can provide transparent and interpretable explanations for their decisions or predictions. In the context of connected healthcare settings, XAI not only helps ensure the quality and safety of care but also fosters trust among patients and healthcare providers. Several notable XAI techniques include: *SHAP (SHapley Additive exPlanations)* values provide a unified framework for explaining the output of any machine learning model by attributing contributions of each input feature to the model’s prediction [29, 30]. *LIME (Local Interpretable Model-Agnostic Explanations)* generates local explanations by approximating complex model behaviour with simpler, interpretable models on a subset of data points [31]. *Saliency Maps* highlight regions in input data (e.g., medical images) that are most influential in a model’s prediction, aiding clinicians in understanding what the model is focusing on [32]. *Accumulated Local Effects (ALE)* helps visualize how the relationship between a single feature and the model’s prediction changes across different feature values [33]. *Contrastive Explanation Method (CEM)* generates contrastive explanations, highlighting the minimal changes needed in input features to alter a model’s prediction, which can be invaluable in understanding model behaviour [34]. *Global Interpretation via Recursive Partitioning (GIRP)* uses recursive partitioning techniques to create a global interpretable model that approximates the original complex model [35]. *CAM (Class Activation Maps) *highlights important regions in images that contribute to a specific class prediction, making it useful for image classification tasks [36]. *GradCAM (Gradient-weighted Class Activation Mapping)* combines gradient information with CAM to provide more precise visualizations of feature importance in convolutional neural networks [37]. *LRP (Layer-wise Relevance Propagation)* is a method that assigns relevance scores to each input feature, explaining how each feature contributes to the model’s output [38]. In this paper, we choose GradCAM and LRP due to their ability to provide precise, visual, and deep-level explanations, their compatibility with CNN-based models, and their established utility in the medical imaging domain. These methods collectively offer a comprehensive solution for improving the interpretability of AI models in a clinical context, ultimately leading to more informed and confident clinical decision-making. The results of each technique are explained as follows:

### GradCAM heatmaps

**Fig. 4:**
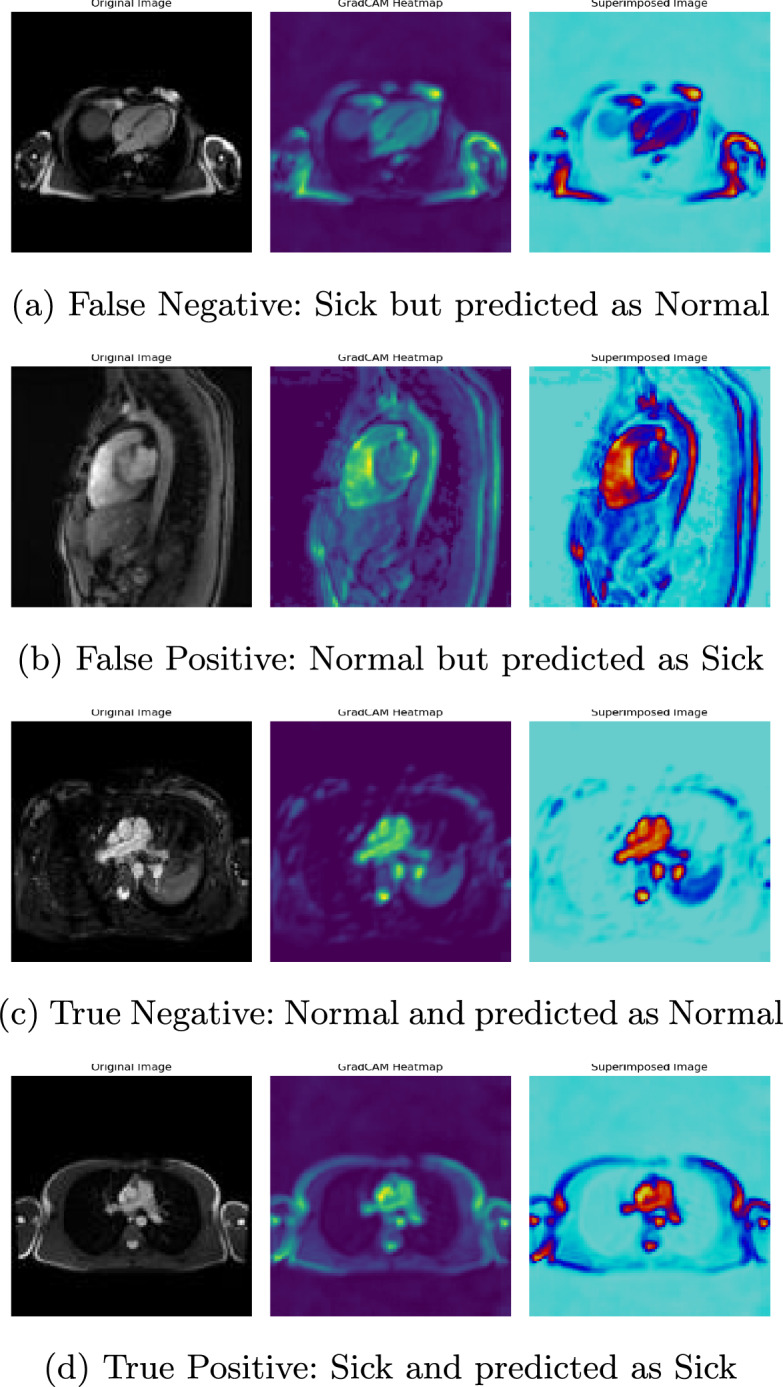
Heatmaps generated by GradCAM on test images. The most important features of the images that contribute to the classification of the image into certain classes are shown in darker colours. The three images are original, heatmap, and superimposed image

Gradient-weighted Class Activation Mapping (Grad-CAM) is a computer vision technique used to generate a heatmap of the important regions in an image that significantly contributes to the prediction of the deep learning model [39]. Figure 4 illustrates some examples of generated GradCAM heatmaps that highlight the focused regions (regions of interest) for the prediction of CAD in the test images. In the GradCAM visualization, the intensity of the heatmap represents the importance of each pixel in the input image. Higher intensity (e.g. brighter colours) and high-contrast colour with the background are indicative of a more significant region that contributed to the model’s prediction.

### Layer-wise relevance propagation (LRP)

Layer-wise Relevance Propagation (LRP) is an XAI technique used to understand the predictions made by deep learning models. The primary objective of LRP is to ascribe the model’s predictions to specific regions or features within the input image [40]. This helps explain why a particular classification decision was made, which is crucial in medical applications for trust and accountability. The core principle shared among various versions of the LRP algorithm is the conservation of the activation strength of an output node for a specific class, as it is propagated back through each layer of the neural network. This ensures that the total relevance associated with a particular class remains constant as it traverses the network layers during the explanation process [41]. This study investigated two versions of the LRP algorithms i.e., LRP0 and LRP_epsilon. The LRP0 is a straightforward version that conserves relevance strictly but can lead to issues with non-differentiable activation functions. LRP_epsilon addresses these issues by introducing a small smoothing factor (epsilon) to improve the stability and interpretability of relevant heatmaps.

**Fig. 5:**
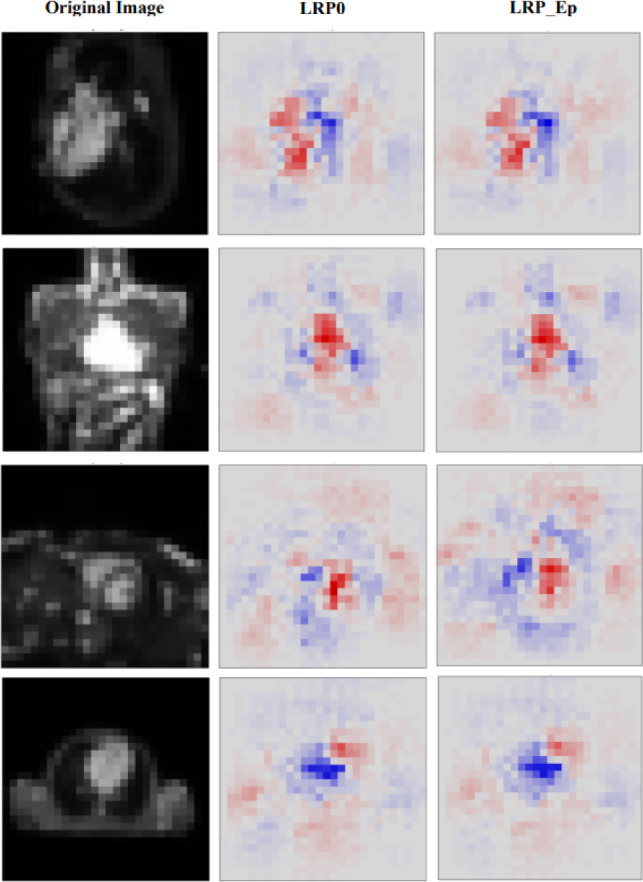
Heatmaps generated by LRP on test images. The left column has original images, the middle column is the output heatmaps of LRP0 while the right column is the output heatmaps of LRP_Epsilon Technique

Figure 5 displays the heatmaps produced by both algorithms along with the original images. The significance of features is visually represented using colours, with red indicating more critical features contributing to the classification of an image into a specific category.

### Failure cases

The lack of contrast in the region of interest (ROI) or overly bright regions where there is no relevant information (ROI) presents a significant challenge. While the model appropriately emphasizes brighter regions, it struggles when the input image does not have enough contrast. Thus, the performance of the proposed model significantly depends on the quality of the input image. To address this issue, a potential solution is to implement a preprocessing step focused on enhancing image contrast. Furthermore, an iterative refinement process and parameter tuning may be employed to optimize the preprocessing step, ensuring adaptability to varying degrees of contrast in input images. However, it is crucial to acknowledge that these approaches incur computational expenses due to the additional processing requirements. Therefore, a trade-off balance between computational resources and enhanced model performance needs to be met.

**Table 6:** Comparison of the proposed model with AlexNet in terms of classification accuracy, model complexity and run-time complexity on 323 test images

Model	Accuracy	Balance accuracy	Inference time (sec)	Trainable parameters	Model size (MB)
AlexNet	98.89%	98.85%	5.10	24708481	94.26
**Our**	**99.35%**	**99.13%**	**2.6**	**507299**	**1.94**

## Conclusion

In conclusion, this research study aimed to propose a light-weighted Convolutional Neural Network (CNN) model tailored for real-time CAD image classification tasks in connected healthcare environments. The study placed a strong emphasis on optimizing hyperparameter configurations to enhance the efficiency and accuracy of AI models in healthcare-related classifications. Moreover, to provide the interpretability of the model’s predictions, we incorporated the GradCam and LRP algorithms, that highlighted the significant features within input images that influence classification decisions.

The achieved results are compared with the state-of-the-art algorithm present in the literature (an ensemble of 10 CNN-RF networks). The CNN-RF model is more computationally expensive as it extracts classification features using CNN and then feeds them to Random Forest (RF) classifier for classification. In addition, majority voting is performed to predict the final class (normal or sick patient image). On the other hand, the proposed model is a single seven-layered CNN model which outperforms the CNN-RF in terms of classification as well as time complexity making it more suitable to be implemented on edge devices and in connected healthcare settings.

The classification model performance (for both the models i.e., baseline and proposed) was measured using PPV, recall, specificity, F1-Score, AUC, and accuracy matrices. As the dataset has a class imbalance, an additional performance metric i.e., Balance Accuracy was also calculated during the analysis. The combination of different hyperparameters revealed different classification accuracies, as tabulated in Table 1 to 5. Among all the settings, the proposed model achieved the highest test accuracy of 99.35% (with balanced accuracy = 99.13%) with interlayer activation function to be PReLU, RMSprop optimizer, batch size of 32 and binary-cross-entropy loss-function.

The proposed model is also compared with a relatively more complex AlexNet in terms of classification accuracy, model complexity, and run-time complexity. With AlexNet achieving an accuracy of 98.89%, the proposed model demonstrates superior performance, as shown in Table 6. In addition to accuracy, the proposed model exhibits substantially reduced training and inference times (556.4 seconds and 2.6 seconds, respectively) compared to AlexNet. Moreover, the architecture of the proposed model has significantly fewer trainable parameters (507,299) as well as a smaller model size (1.94 MB), demonstrating its enhanced practicality and resource efficiency.

The achievement of such a high classification accuracy on the CAD test dataset with downsized images (100x100 pixels) using the proposed light-weighted model can be attributed to two main factors. Firstly, the representational efficiency of the model architecture is a key contributor. The proposed model demonstrates the capacity to learn the crucial features even in low-resolution images, enabling accurate predictions. Secondly, the downsampling of images does not severely compromise the model’s proficiency in recognizing spatial hierarchies and patterns.

This research highlights the critical role of optimizing time complexity and hyperparameters in the development of sustainable healthcare AI models. By doing so, we can ensure the resource efficiency and real-time applicability of these models, while concurrently upholding their reliability. Furthermore, the incorporation of eXplainable AI (XAI) techniques provides essential interpretability, aligning AI-generated recommendations with the interpretations of clinical experts and safeguarding patient safety.

***Future directions:*** The proposed investigative work aimed to provide insight into the optimization of healthcare AI models, ensuring accurate and reliable results while prioritizing patient safety, resource efficacy, and advancing the acceptance and understanding of AI in connected healthcare settings. While the results on the 2D CMR images are promising, in future 3D-CNN based models will be explored on other healthcare images such as Computer Tomography (CT), X-rays and/or Echocardiogram (Echos) images to determine the model’s comprehensive diagnostic capabilities, cross-domain scalability, and performance on Multi-model data. Moreover, we propose the integration of two techniques to further improve the designed classification models’ performances: majority voting for frame-based analysis and the implementation of a video-based classifier. Combining these techniques offers a promising path towards a more accurate and reliable classification model to distinguish between patient and healthy images in MRI scans.

## Footnotes

1 https://www.who.int/news-room/fact-sheets/detail/cardiovascular-diseases-%28cvds%29.

2 https://www.kaggle.com/danialsharifrazi/cad-cardiac-mri-dataset.

3 https://d2l.ai/chapter_convolutional-neural-networks/lenet.html.
